# Neuropeptide Y inhibits interleukin-1β-induced phagocytosis by microglial cells

**DOI:** 10.1186/1742-2094-8-169

**Published:** 2011-12-02

**Authors:** Raquel Ferreira, Tiago Santos, Michelle Viegas, Luísa Cortes, Liliana Bernardino, Otília V Vieira, João O Malva

**Affiliations:** 1 Center for Neuroscience and Cell Biology, University of Coimbra, Coimbra, Portugal; 2 Laboratory of Biochemistry and Cell Biology, Faculty of Medicine, University of Coimbra, Coimbra, Portugal

**Keywords:** microglia, neuropeptide Y, HSP27, p38, inflammation, phagocytic cups

## Abstract

**Background:**

Neuropeptide Y (NPY) is emerging as a modulator of communication between the brain and the immune system. However, in spite of increasing evidence that supports a role for NPY in the modulation of microglial cell responses to inflammatory conditions, there is no consistent information regarding the action of NPY on microglial phagocytic activity, a vital component of the inflammatory response in brain injury. Taking this into consideration, we sought to assess a potential new role for NPY as a modulator of phagocytosis by microglial cells.

**Methods:**

The N9 murine microglial cell line was used to evaluate the role of NPY in phagocytosis. For that purpose, an IgG-opsonized latex bead assay was performed in the presence of lipopolysaccharide (LPS) and an interleukin-1β (IL-1β) challenge, and upon NPY treatment. A pharmacological approach using NPY receptor agonists and antagonists followed to uncover which NPY receptor was involved. Moreover, western blotting and immunocytochemical studies were performed to evaluate expression of p38 mitogen-activated protein kinase (MAPK) and heat shock protein 27 (HSP27), in an inflammatory context, upon NPY treatment.

**Results:**

Here, we show that NPY inhibits phagocytosis of opsonized latex beads and inhibits actin cytoskeleton reorganization triggered by LPS stimulation. Co-stimulation of microglia with LPS and adenosine triphosphate also resulted in increased phagocytosis, an effect inhibited by an interleukin-1 receptor antagonist, suggesting involvement of IL-1β signaling. Furthermore, direct application of LPS or IL-1β activated downstream signaling molecules, including p38 MAPK and HSP27, and these effects were inhibited by NPY. Moreover, we also observed that the inhibitory effect of NPY on phagocytosis was mediated *via *Y_1 _receptor activation.

**Conclusions:**

Altogether, we have identified a novel role for NPY in the regulation of microglial phagocytic properties, in an inflammatory context.

## Background

Microglia are the resident immunocompetent cells of the central nervous system (CNS), responsible for mounting appropriate responses to injuries such as trauma, ischemia, brain tumors and neurodegenerative diseases that target the brain parenchyma [1,2]. Moreover, microglia display a wide range of receptors that enable the recognition of pathogens or cell damage-related antigens, thereby promoting phagocytosis and removal of cell debris [3]. Phagocytosis is a coordinated process, triggered by environmental signals that requires a dynamic actin cytoskeleton rearrangement and a plethora of receptor signaling pathways [4].

The role of microglia in inflammation has been experimentally dissected using lipopolysaccharide (LPS) stimulation, which mimics Gram-negative infection, through the activation of Toll-like receptor 4 (TLR4). While microglia randomly scan the healthy brain parenchyma, activated cells undergo significant morphological changes and an ensuing targeted movement toward the site of injury, where they release both neurotrophic and neurotoxic factors [2,5]. Amongst the inflammatory mediators initially secreted, interleukin-1β (IL-1β) is particularly relevant given its involvement in excitotoxicity, ischemia, brain trauma and cell death [6-8]. Recently, we have described a chemokinetic effect of IL-1β on microglial motility, whereby IL-1β stimulates microglial motility with involvement of p38 MAPK signaling [9].

Growing evidence supports involvement of neuropeptide Y (NPY) in the modulation of the immune system, with effects on macrophage, B and T cell function; as well as dendritic cell stimulatory ability [10]. However, its role in phagocytosis remains controversial. Neuropeptide Y (NPY) is widely distributed within the peripheral and central nervous systems and has well defined physiological roles that include regulation of blood pressure, circadian rhythms, feeding behavior, memory processing and learning [11].

In this context, our objective was to unravel the role of NPY in the modulation of Fc receptor-mediated phagocytosis (the best characterized phagocytic receptor) by activated microglial cells during inflammation. Microglial cells have specific signaling systems that regulate rapid rearrangement of the actin cytoskeleton enabling the cell to phagocytose when needed. Here, we report an inhibitory effect of NPY, acting *via *Y_1 _receptors, on IL-1β-stimulated phagocytosis, a process accompanied by p38 MAPK and HSP27 activation. Our results highlight the modulation of phagocytosis as part of the putative anti-inflammatory role of NPY, supporting the importance of this neuropeptide in the regulation of important microglial responses to danger signals in the brain.

## Methods

### Cell line cultures

A murine N9 microglia cell line (a kind gift from Professor Claudia Verderio, CNR Institute of Neuroscience, Cellular and Molecular Pharmacology, Milan, Italy) was grown in RPMI medium supplemented with 30 mM glucose (Sigma, St. Louis, MO, USA), 100 U/ml penicillin and 100 μg/ml streptomycin (GIBCO, Invitrogen, Barcelona, Spain). Cells were kept at 37°C in a 95% atmospheric air and 5% CO_2 _humidified atmosphere. Numbers of viable cells were evaluated by counting trypan blue-excluding cells that were then plated at a density of 2 × 10^4 ^cells per well in 24-well trays, or plated at a density of 5 × 10^5 ^cells per well in 6-well trays (for remaining experiments).

Cell treatment for phagocytosis studies included the following incubation setup: NPY (human, rat/amidated sequence) (1 μM) (Bachem, Bubendorf, Switzerland), LPS (100 ng/ml) (Sigma), ATP (1 mM) (Sigma), IL-1β (1.5 ng/ml) (R&D System, Minneapolis, MN, USA), IL-1ra (150 ng/ml) (R&D Systems), SB239063 (chemically synthesized) (20 μM) (Tocris, Bristol, UK), Y_1 _receptor agonist [Leu31, Pro34]NPY (porcine, amidated sequence) (1 μM) (Bachem), Y_1 _receptor antagonist BIBP3226 (1 μM, in water) (Bachem), Y_2 _receptor antagonist BIIE0246 (1 μM, in 0.06% DMSO) (Tocris) and Y_5 _receptor antagonist L-152,804 (1 μM, in 0.2% DMSO) (Tocris), for 6 hrs. ATP, SB239063 and all receptor antagonists were added 30 min prior to cell treatment and maintained during the course of experiments.

### Generation of an N9 murine microglia cell line stably expressing human FcγRIIA

The N9 microglia cell line expresses a variety of Fc receptors. To overcome this issue, and due to the absence of good antibodies against Fc receptors, we decided to constitutively express human FcγRIIA tagged with c-myc. The protocol for generation of N9 cells expressing FcγRIIA, retrovirus production, cell infection and selection was adopted from [12,13]. Briefly, human FcγRIIA tagged with C-terminal myc-His6 was subcloned into the retroviral vector pBABE-puro, which contains a resistance gene to puromycin. The viruses were produced by tranfecting the human Phoenix gag-pol packaging cell line with the retroviral plasmid together with incorporation of the vesicular stomatitis virus G protein. Because of the incorporation of the vesicular stomatitis virus G protein into the virus envelope, these viral particles are able to infect almost any dividing mammalian cell. For infection, 1.5 × 10^5 ^cells were incubated with 1 ml of retrovirus, pseudotyped with the VSV-G envelope protein, expressing the Fc-receptor in the presence of 4 μg/ml polybrene (Sigma), at 32°C. After 24 hrs of incubation, the medium was changed and the infection procedure repeated. Twenty-four hours later, cells were trypsinized and seeded into a 6-well tray in the presence of 7 μg/ml puromycin (Sigma). Selection was done for another 48 hrs.

### Phagocytosis assay

Beads were opsonized with rabbit IgG (1 μg/ml) (Sigma) or with human IgG (0.5 μg/ml) (Sigma, St. Louis, MO, USA) (for the phagocytic cup studies) under constant agitation at 8 rpm, overnight at 4°C. Beads were then resuspended in modified RPMI medium, without NaHCO_3_, and distributed at a density of 1 × 10^5 ^beads per well. After 40 min of incubation (or 20 min, for phagocytic cup studies), cells were washed with PBS and fixed with 4% paraformaldehyde (PFA). Extracellular and/or adherent beads were labeled with secondary antibody Alexa Fluor 594 donkey anti-rabbit or with Cy5 donkey anti-human (Molecular Probes, Oregon, USA), 1: 500, in PBS. For each field, three photomicrographs were acquired in order to capture stained nuclei (in blue), extracellular and/or adherent beads (in red) and total number of beads (differential interference contrast image). The location of each bead was analyzed by comparing the three separate images simultaneously. Only beads without fluorescent labeling were considered as internalized particles. For nuclear labeling, cell preparations were stained with Hoechst 33342 (2 μg/ml) (Molecular Probes) in PBS, for 5 min at room temperature (RT). Coverslips were then mounted in Dako fluorescent medium (Dakocytomation Inc., California, USA). Fluorescent images were acquired using an Axiovert 200 M microscope, equipped with an AxiocamHRm and Plan-ApoChromat 40 ×/1.30 oil objective (Göttingen, Germany).

### Immunocytochemistry

Cells were fixed with 4% PFA (Sigma) and unspecific binding was prevented by incubating cells in a 3% BSA and 0.3% Triton X-100 solution (all from Sigma) for 30 min, at RT. Cells were kept overnight at 4°C, in 0.3% BSA and 0.1% Triton X-100 primary antibody solution, then washed with PBS, and incubated for 1 hr at RT with the corresponding secondary antibody.

Antibodies used were: rabbit polyclonal anti-phosphorylated HSP27 (1:400) (Cell Signaling Tech, Beverly, MA, USA); mouse monoclonal anti-c-myc (1:2000) (Cell Signaling Tech,); rat monoclonal anti-CD11b (1:1000) (AbD Serotec, Oxford, UK); Alexa Fluor 594 goat anti-rabbit; Alexa Fluor 488 donkey anti-rabbit; Alexa Fluor 594 donkey anti-mouse; Alexa Fluor 488 goat anti-rat (all 1:200 in PBS, from Molecular Probes).

Membrane ruffling was observed using a marker for filamentous actin, phalloidin. Cells were incubated for 2 hrs in phalloidin-Alexa Fluor 594 conjugate, 1:100 (Molecular Probes) in PBS, at RT, protected from light.

For nuclear labeling, cell preparations were stained with Hoechst 33342 (2 μg/ml) (Molecular Probes, Eugene, Oregon, USA) in PBS, for 5 min at RT and mounted in Dakocytomation fluorescent medium (Dakocytomation Inc., California, USA). Fluorescent images were acquired using a confocal microscope, with a Plan-ApoChromat 63 ×/1.40 oil objective (LSM 510 Meta, Carl Zeiss, Göttingen, Germany).

### Western blotting

Cells were incubated with lysis cocktail solution (137 mM NaCl, 20 mM Tris-HCl, 1% Triton X-100, 10% glycerol, 1 mM phenylmethylsulfonyl fluoride, 10 μg/ml aprotinin, 1 μg/ml leupeptin, 0.5 mM sodium vanadate (all from Sigma), pH 8.0). After gentle homogenization, the total amount of protein was quantified using the BCA method (Thermo Scientific, Rockford, USA). Afterwards, samples were loaded onto 10% acrylamide/bisacrilamide gels (BioRad, Hercules, CA, USA). Proteins were separated by SDS-PAGE using a bicine/SDS (Sigma) electrophoresis buffer (pH 8.3) and then transferred to PVDF membranes (Millipore) with a 0.45-μm pore size, under the following conditions: 300 mA, 90 min at 4°C in a solution containing 10 mM CAPS (Sigma) and 10% methanol (VWR International S.A.S. France), pH 11.0) (protocol adapted from [14]). For detection of phosphorylated proteins, membranes were blocked in Tris-buffer saline (TBS) containing 5% BSA, 0.1% Tween^® ^20 (Sigma) for 1 hr, at RT, and then incubated overnight at 4°C with the primary antibody diluted in blocking solution.

The following primary antibodies were used: rabbit polyclonal anti-phosphorylated HSP27 (1:1000), rabbit polyclonal anti-HSP27 (1:1000) (both from Cell Signaling) and mouse monoclonal anti-c-myc (1:600) (Santa Cruz Biotechnology Inc.). After rinsing three times with TBS-T, membranes were incubated for 1 hr at RT with an alkaline phosphatase-linked secondary antibody anti-rabbit IgG 1:20,000 or anti-mouse IgG 1:10,000 in blocking solution (GE Healthcare UK Limited, Buckinghamshire, UK). Protein immunoreactive bands were visualized in a Versa-Doc Imaging System (Model 3000, BioRad Laboratories, CA, USA), after incubation of the membranes with enhanced chemofluorescence reagent (GE Healthcare UK Limited) for 5 min.

### Data analysis

Statistical analysis was performed using GraphPad Prism 5.0 (GraphPad Software, San Diego, CA). Statistical significance was considered relevant for *p *values < 0.05 using one-way analysis of variance followed by Bonferroni *post hoc *test for comparison among experimental settings and Dunnett *post hoc *test for comparison with control condition. Data are presented as mean ± standard error of mean (SEM). For every immunocytochemical analysis, 5 independent microscopy fields were acquired per coverslip (about 50 cells per field). Every experimental condition was tested in three sets of independent experiments (*n*), unless stated otherwise, and performed in duplicate.

## Results

### NPY inhibits Fc receptor-mediated phagocytosis by microglial cells

The murine N9 microglia cell line was used to discern the role of NPY in LPS-induced phagocytosis. LPS is a component of Gram-negative bacterial outer membranes and binds to the CD14/TLR4/MD2 receptor complex present at the microglial cell membrane, triggering several signaling cascades [15]. We have previously used this cell line to dissect the effects of LPS over other microglial physiological responses, such as production of inflammatory mediators [e. g. nitric oxide (NO) and IL-1β)] and migration/motility. We observed that NPY, acting *via *the Y_1 _receptor, inhibited LPS-induced microglial activation [9,16].

**Figure 1:**
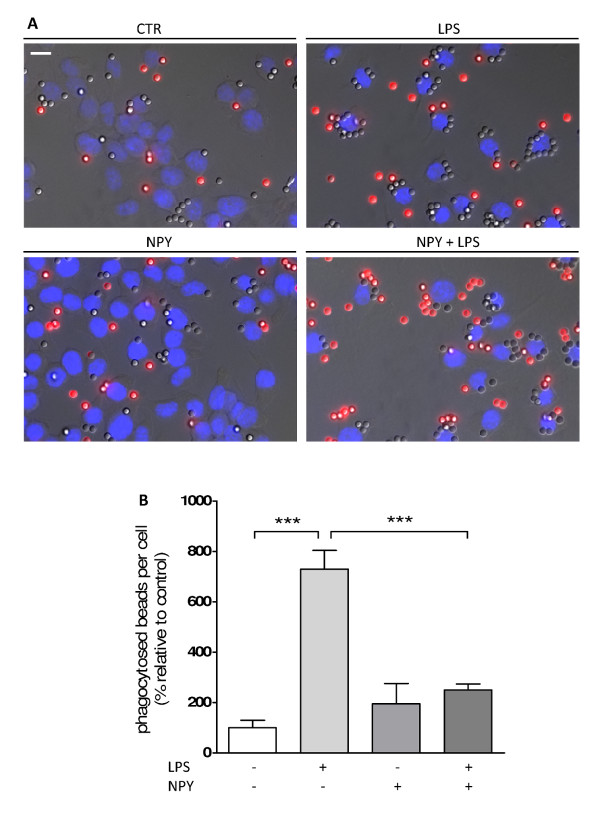
**NPY inhibits bead phagocytosis by microglial cells**. (A) Representative photomicrographs illustrate the inhibitory effect of NPY on LPS-induced phagocytosis. (B) LPS (100 ng/ml) increased bead phagocytosis, while NPY (1 μM) inhibited this effect. Data are expressed as mean ± SEM (n = 3-5) and as a percentage of control (***p < 0.001, using Bonferroni's Multiple Comparison Test). Scale bar 10 μm.

Prior to phagocytosis, microglial cells were challenged with LPS (100 ng/ml) and/or NPY (1 μM) for 6 hrs. IgG-opsonized latex beads were added at a density of 1 × 10^5 ^per well and allowed to be ingested for 40 min. After fixation, non-ingested beads were immunolabeled in order to distinguish extracellular and adherent particles from those internalized. Therefore, phagocytosed beads were distinguished from non-phagocytosed beads by means of fluorescent labeling (none versus red, respectively) (Figure 1A). LPS significantly increased bead phagocytosis, while NPY inhibited this effect (mean_CTR _= 100 ± 29.46%; mean_LPS _= 730.50 ± 74.02%; mean_LPS+NPY _= 250 ± 23.47%; mean_NPY_= 195.60 ± 79.83%; p < 0.001) (Figure 1B). In Figure 1A, representative photomicrographs illustrate the inhibitory effect of NPY over LPS-stimulated microglia phagocytosis.

### LPS-induced phagocytosis involves IL-1β signaling

**Figure 2:**
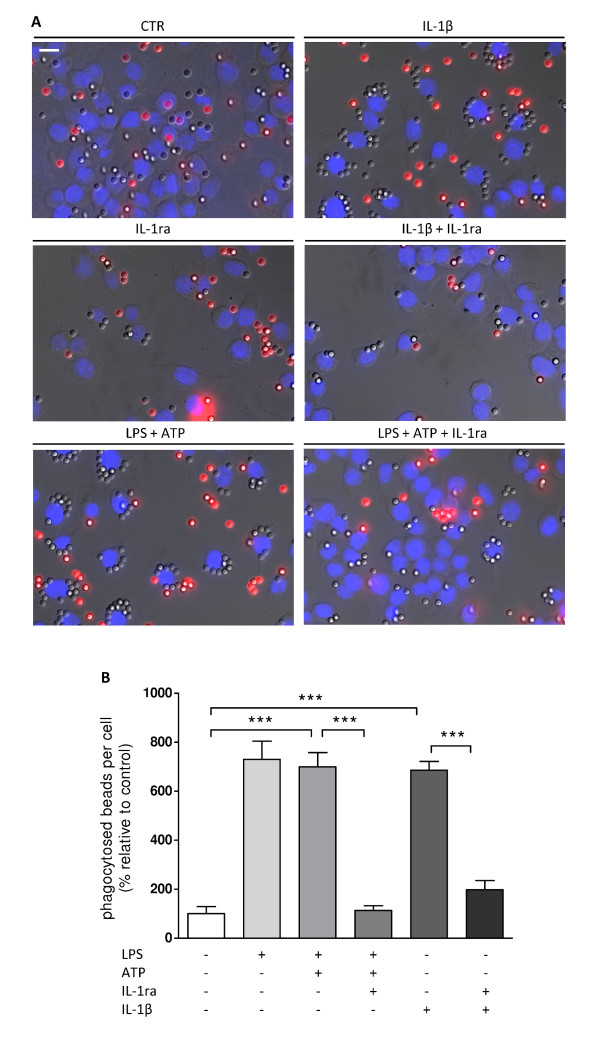
**LPS-induced phagocytosis is mediated by IL-1β signaling**. (A) Representative photomicrographs illustrate the inhibitory effect of IL-1β, or LPS+ATP alone or with co-treatment with IL-1ra. (B) LPS (100 ng/ml) and ATP (1 mM) co-administration significantly induced bead phagocytosis. This effect was prevented by IL-1ra application (150 ng/ml) suggesting involvement of IL-1β. Direct application of IL-1β (1.5 ng/ml) increased phagocytosis and was completely inhibited by IL-1ra. Data are expressed as mean ± SEM (n = 3-5) and as a percentage of control (***p < 0.001, using Bonferroni's Multiple Comparison Test). Scale bar 10 μm.

LPS and ATP co-administration induces a massive release of IL-1β [17-20]. We have previously shown that murine N9 microglial cells release the biologically active form of IL-1β upon LPS and ATP challenge [16]. LPS activates TLR4, triggering several inflammatory responses, while ATP exposure stimulates P2X_7 _receptors, leading to opening of a non-selective pore that allows massive calcium entry and, consequently, activation of interleukin converting enzyme (ICE) [21,22]. In our study, LPS (100 ng/ml) plus ATP (1 mM) significantly stimulated opsonized beads uptake (mean_CTR _= 100 ± 27.91%; mean_LPS+ATP _= 699.50 ± 58.33%; p < 0.001) (Figure 2). Interestingly, this effect was completely abolished by interleukin-1β receptor antagonist (IL-1ra) treatment (150 ng/ml), suggesting involvement of IL-1β in LPS-plus-ATP-induced microglial phagocytosis (mean_LPS+ATP _= 699.50 ± 58.33%; mean_LPS+ATP+IL-1ra _= 113.90 ± 19.02%; p < 0.001). Accordingly, IL-1β (1.5 ng/ml) significantly increased microglial cell phagocytosis, which was also inhibited by exposure to IL-1ra (mean_IL-1β _= 685.90 ± 36.37%; mean_IL-1β+IL-1ra _= 198 ± 37.10%; p < 0.001) (Figure 2B). In Figure 2A, representative photomicrographs illustrate a stimulatory effect of IL-1β on microglial phagocytosis, as well as LPS and ATP, and an inhibitory effect of IL-1ra over these events.

### NPY inhibits IL-1β-stimulated phagocytosis *via *Y_1 _receptor activation

**Figure 3:**
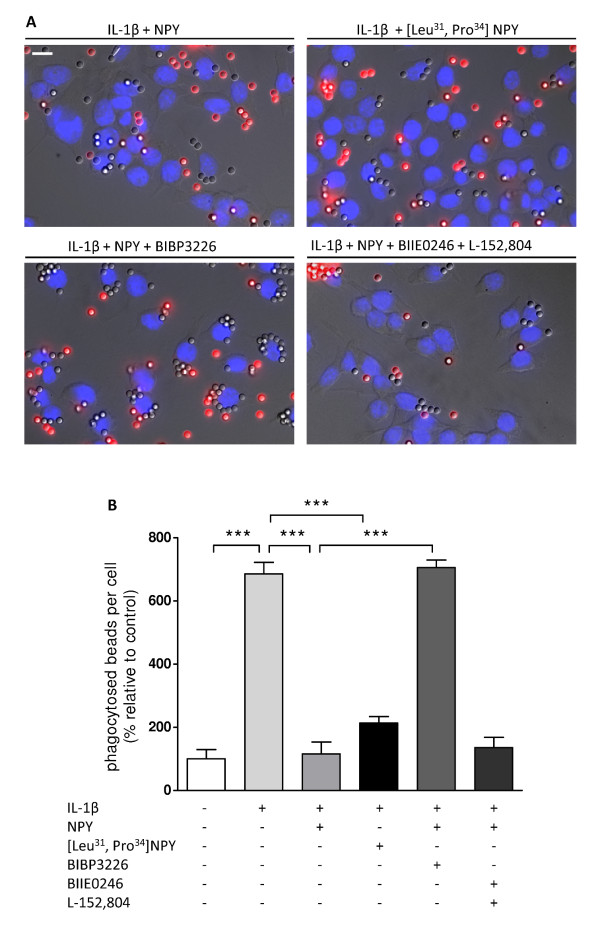
**NPY inhibits IL-1β-induced phagocytosis *via *Y_1 _receptor activation**. (A) Representative photomicrographs illustrate the inhibitory effect of NPY acting *via *Y_1 _receptor on IL-1β-induced cell phagocytosis. (B) Microglial cells were stimulated with IL-1β (1.5 ng/ml) and treated with NPY (1 μM). As NPY inhibited IL-1β-induced phagocytosis, a Y_1 _receptor agonist [Leu^31^, Pro^34^]NPY (1 μM) and a Y_1 _receptor antagonist BIBP3226 (1 μM) were used to determine the effect of Y_1_R activation on IL-1β-induced phagocytosis. The involvement of other NPY receptors was ruled out with the use of antagonists for the Y_2 _receptor (BIIE0246, 1 μM) and the Y_5 _receptor (L152-804, 1 μM). Data are expressed as mean ± SEM (n = 3-5) and as a percentage of control (***p < 0.001, using Bonferroni's Multiple Comparison Test). Scale bar 10 μm.

In accordance with the previous experiments, microglial cells were then treated with IL-1β (1.5 ng/ml) and NPY (1 μM). As a result, we observed that NPY inhibited IL-1β-induced phagocytosis (mean_IL-1β _= 685.90 ± 36.37%; mean_IL-1β+NPY _= 115.40 ± 37.99%; p < 0.001) (Figure 3A and 3B). Moreover, to assess through which receptor NPY inhibits microglial phagocytic activity, cells were treated with the Y_1 _receptor agonist [Leu^31^, Pro^34^] NPY (1 μM) or the Y_1 _receptor antagonist BIBP3226 (1 μM). Y_1 _receptor activation resulted in inhibition of IL-1β-induced phagocytosis while the Y_1 _receptor antagonist blocked the effect induced by NPY (mean_IL-1β+[Leu, Pro]NPY _= 213.70 ± 20.37%; mean_IL-1β+NPY+BIBP3226 _= 705.90 ± 23.89%; p < 0.001). Involvement of the other main NPY-stimulated receptors in brain was excluded using a combination of selective antagonists for Y_2 _receptor (BIIE0246, 1 μM) and for Y_5 _receptor (L152-804, 1 μM), since, in the presence of both antagonists, NPY was still able to inhibit phagocytosis (mean_IL-1β+NPY+BIIE0246+L152-804 _= 135.70 ± 32.65%) (Figure 3A and 3B).

### LPS-stimulated phagocytosis requires p38 signaling

**Figure 4:**
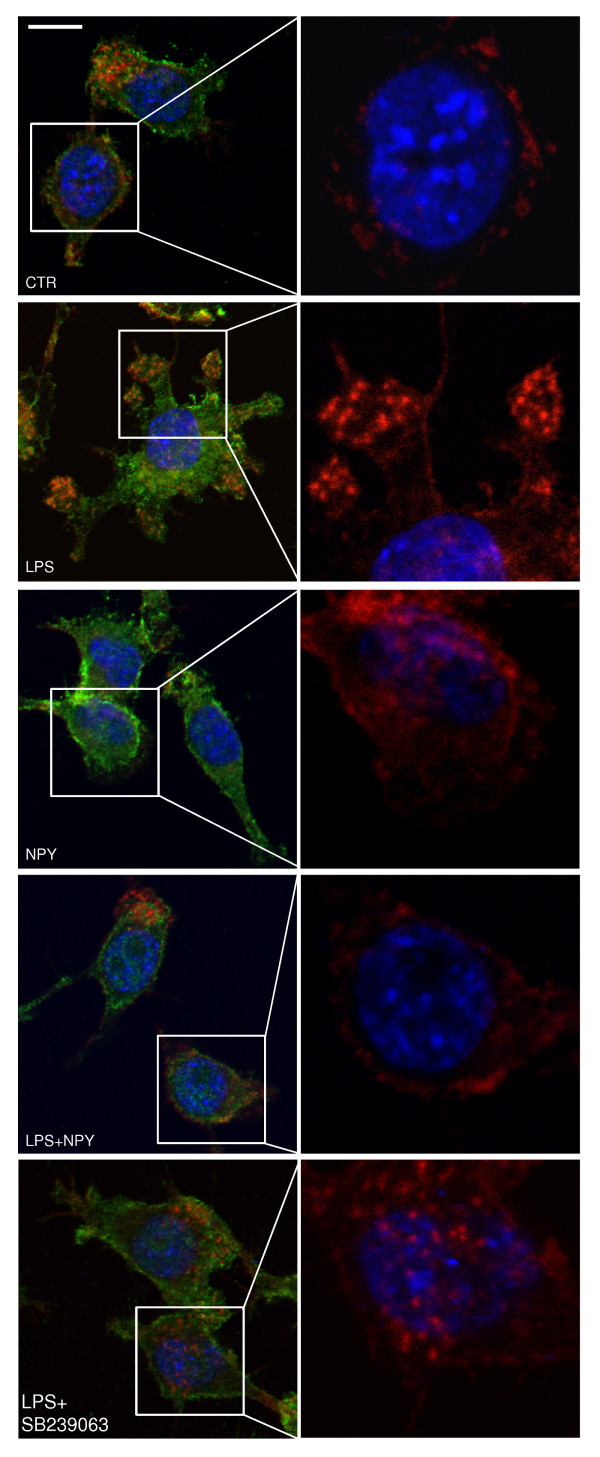
**NPY inhibits LPS-induced actin cytoskeleton reorganization**. Representative confocal photomicrographs were taken to assess the role of LPS on rearrangement of the actin cytoskeleton. Microglial cells treated with LPS (100 ng/ml) showed significant membrane ruffling, while NPY (1 μM) reduced this effect. Moreover, microglial cells treated with LPS (100 ng/ml), in the presence of SB239063, showed a cytoskeleton rearrangement similar to that of untreated cells. Cells were stained for actin (in red), CD11b (in green) and Hoechst 33342 (nuclei in blue). Scale bar 10 μm.

We have previously observed that p38 MAPK signaling pathway is activated during actin cytoskeletal assembly. Moreover, p38 inhibition dampens this process. Accordingly, we hypothesized that p38 was involved in phagocytosis, a very well coordinated process that requires dynamic arrangement of actin filaments [4]. For that matter, we decided to investigate whether the NPY inhibitory effect on phagocytosis is caused by any visible alterations in the reorganization of the actin cytoskeleton, using phalloidin staining. Cell morphology was assessed using the labeling with the surface marker CD11b, which is expressed by microglial cells [23] (Figure 4). Upon LPS challenge, actin (in red) actively polymerized and formed exuberant and flexible structures named membrane ruffles; this feature can contribute, at least in part, to the increased rate of phagocytosis. Untreated cells or cells otherwise treated with NPY mainly exhibited cortical actin. We would like to stress that there was a huge reduction in membrane ruffles on NPY-treated cells compared with LPS-treated cells (Figure 4). Thus, the effect of NPY on phagocytosis upon LPS challenge can be attributed, at least partially, to changes in actin cytoskeleton rearrangements. Accordingly, in the presence of the p38 inhibitor SB239063 (20 μM), actin cytoskeleton morphology resembled that of untreated or NPY-treated cells (Figure 4).

**Figure 5:**
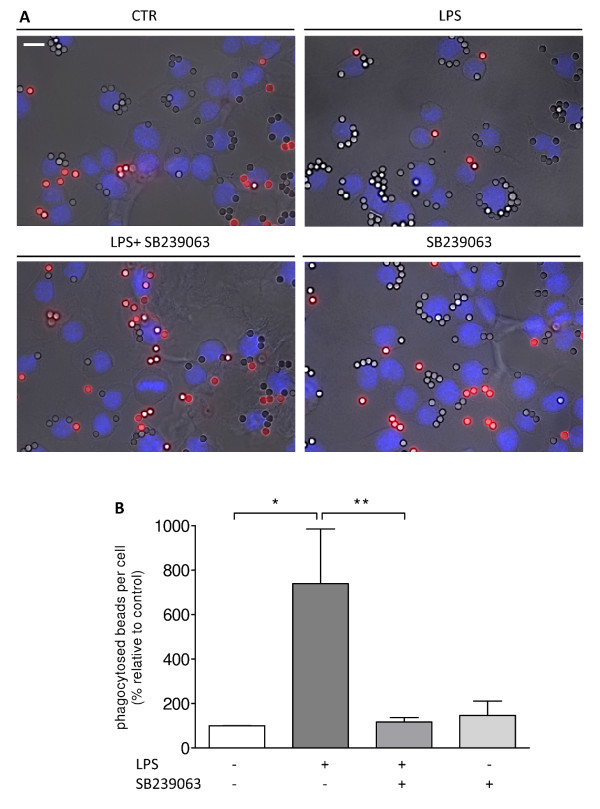
**LPS-stimulated phagocytosis requires p38 activation**. (A) Representative photomicrographs depict involvement of p38 signaling in LPS-induced phagocytosis. (B) Microglial cells were treated with LPS (100 ng/ml). Application of a p38 inhibitor, SB239063 (20 μM), significantly inhibited LPS-induced phagocytosis. Data are expressed as mean ± SEM (n = 3) and as a percentage of control (*p < 0.05; **p < 0.01, using Bonferroni's Multiple Comparison Test). Scale bar 10 μm.

**Figure 6:**
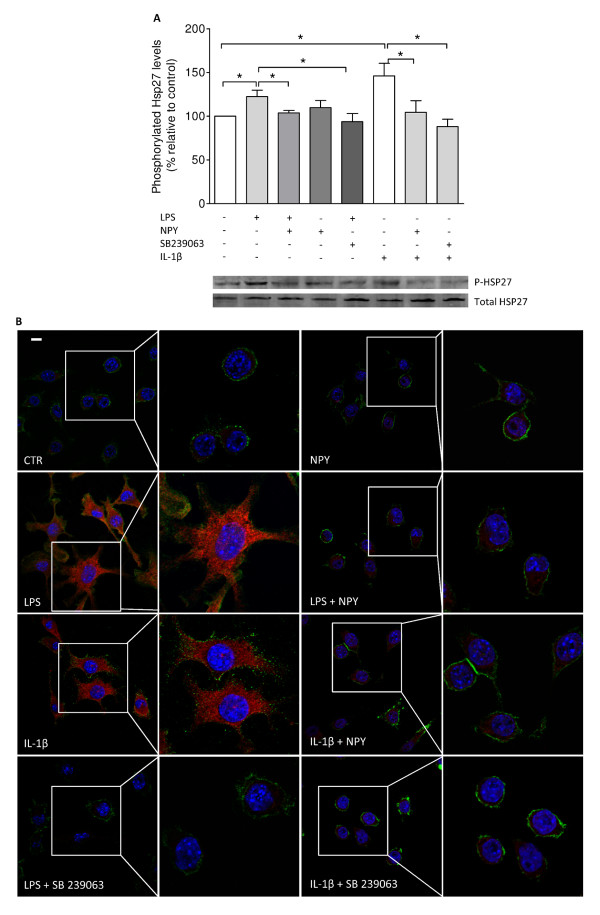
**NPY inhibits LPS- and IL-1β-induced HSP27 phosphorylation**. (A) Densitometric quantification of western blots shows that microglial cells, treated with LPS (100 ng/ml) or with IL-1β (1.5 ng/ml) showed increased levels of phosphorylated HSP27, while NPY (1 μM) clearly inhibited this effect. Moreover, cells treated with the p38 inhibitor SB239063 (20 μM) showed decreased expression similar to that of untreated cells. (B) Representative confocal photomicrographs demonstrate increased phosphorylation of HSP27 upon inflammatory challenge. Cells were stained for phosphorylated HSP27 (in red), CD11b (in green) and Hoechst 33342 (nuclei in blue). Data are expressed as mean ± SEM (n = 4-5) and as a percentage of control (*p < 0.05, using Bonferroni's Multiple Comparison Test). Scale bar 10 μm.

In addition, we observed that in the presence of the p38 inhibitor, LPS-stimulated phagocytosis was reduced and was similar to untreated cells (mean_CTR _= 100 ± 0.01%; mean_LPS _= 740.5 ± 246%; mean_LPS+SB239063 _= 118 ± 19.45%; mean_SB239063 _= 147.1 ± 63.81%; p < 0.05) (Figure 5A and 5B). Heat shock protein 27 (HSP27) is a downstream target of p38, also implicated in the control of actin polymerization [24]. Herein, we observed by western blot and immunocytochemistry that both LPS and IL-1β significantly induced HSP27 phosphorylation (mean_CTR _= 100%; mean_LPS _= 122.5 ± 7.25%; mean_IL-1β _= 146.1 ± 14.35%; p < 0.05), while a p38 inhibitor (SB239063) inhibited this effect (mean_LPS+SB239063 _= 93.8 ± 9.21%; mean _IL-1β+SB239063 _= 88.17 ± 8.48%; p < 0.05), supporting the involvement of p38 signaling in HSP27 activation (Figure 6). Additionally, NPY inhibited LPS- and IL-1β-induced HSP27 phosphorylation (mean_LPS+NPY _= 103.8 ± 2.74%; mean _IL-1β+NPY _= 104.3 ± 13.58%; mean_NPY _= 109.9 ± 8.13%; p < 0.05) (Figure 6A). In Figure 6B, HSP27 labeling (in red) is significantly increased in LPS- and IL-1β-activated cells compared to untreated cells. To visualize cell morphology, we labeled cells with the surface marker CD11b, which is known to be expressed by microglial cells [23].

**Figure 7:**
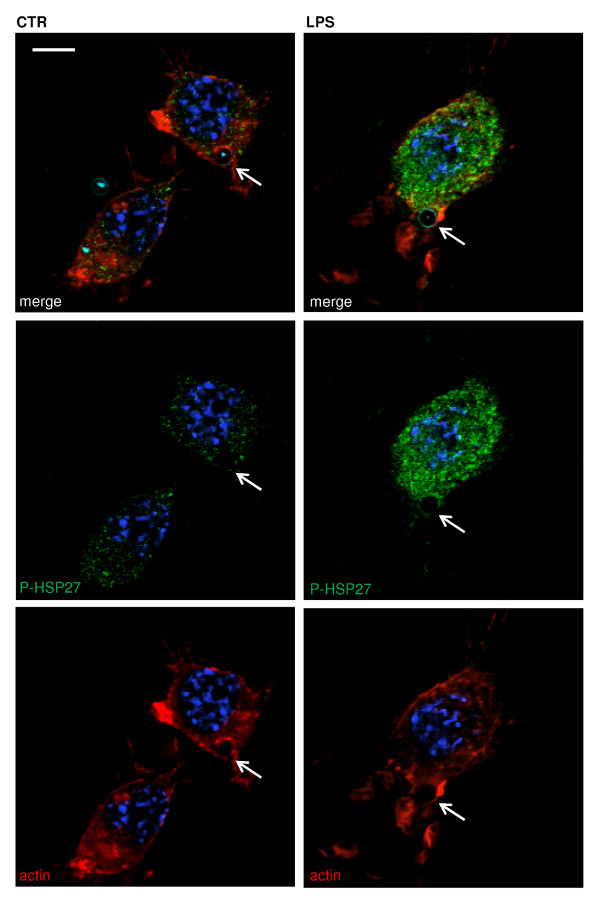
**HSP27 is present at the phagocytic cup**. Representative confocal photomicrographs were taken to assess the role of LPS on formation of the phagocytic cup. Untreated microglial cells (left panel) and cells treated with LPS (100 ng/ml) (right panel) revealed formation of a cup-shaped structure engulfing an opsonized bead (open arrowhead). LPS treatment induced increased labeling of phosphorylated HSP27 throughout the cell cytoplasm and nucleus as well as in the cup. Cells were stained for phosphorylated HSP27 (in green), actin (in red) and Hoechst 33342 (nuclei in blue). Opsonized beads are labeled in cyan blue. Scale bar 10 μm.

Interestingly, in accordance with the increased expression of phosphorylated p38 and HSP27 in LPS or IL-1β-treated cells, we also observed that HSP27 co-localized with the actin cup surrounding beads in the process of phagocytosis. HSP27 expression in the phagocytic cups observed in LPS-treated cells was clearly potentiated when compared to phagocytic cups in non-stimulated cells (showing a faint but still present staining of HSP27) (Figure 7).

### NPY reduces FcγRIIA expression on LPS- and IL-1β-stimulated microglia

**Figure 8:**
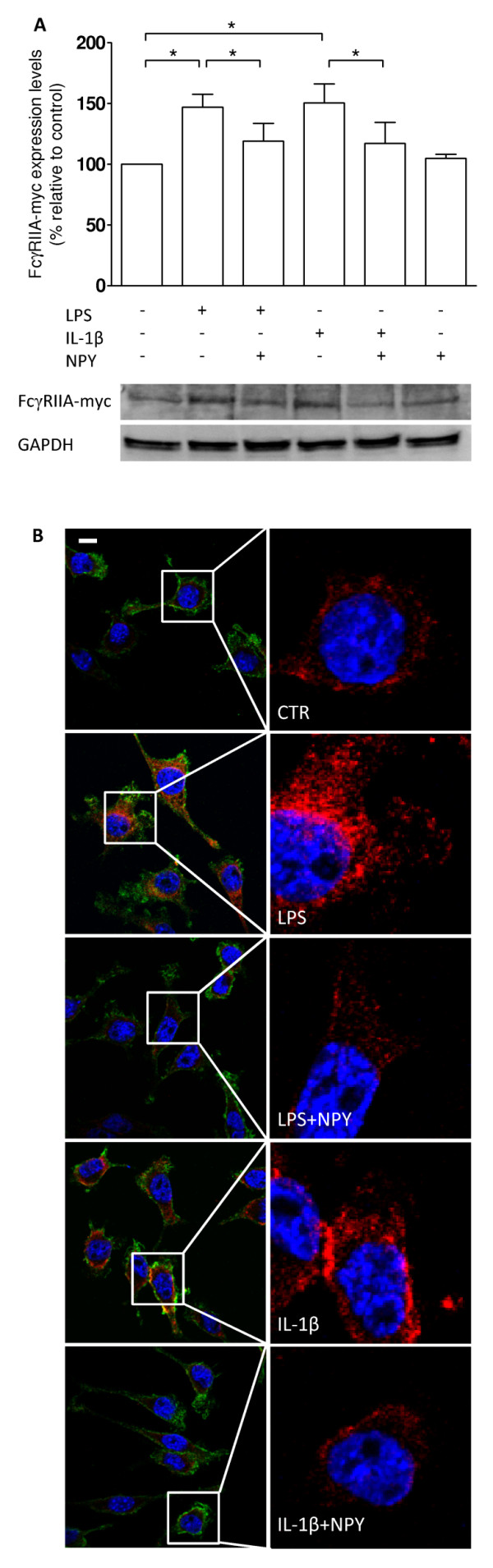
**NPY decreases FcγRIIA-myc expression on microglial cells**. (A) Densitometric quantification of western blots shows that microglial cells treated with LPS (100 ng/ml) or with IL-1β (1.5 ng/ml) increased FcγRIIA expression levels while NPY treatment (1 μM) significantly inhibited this effect. (B) Representative confocal photomicrographs demonstrate an inhibitory effect of NPY on LPS- or IL-1β-induced FcγRIIA expression. Cells were stained for FcγRIIA-myc (in red), CD11b (in green) and Hoechst 33342 (nuclei in blue). Data are expressed as mean ± SEM (n = 4) and as a percentage of control (*p < 0.05, using Bonferroni's Multiple Comparison Test). Scale bar 10 μm.

To further evaluate the role of NPY on FcR-mediated phagocytosis, we transfected N9 microglial cells in order to stably expresses human FcγRIIA, a receptor mainly involved in the phagocytosis of opsonized particles [25]. In that sense, we observed that upon LPS or IL-β challenge, the derived N9 microglia cell line significantly increased FcγRIIA protein levels, quantified by western blotting (mean_CTR _= 100 ± 0.01%; mean_LPS _= 128.30 ± 7.13%; mean_IL-1β _= 123.70 ± 7.69%; n = 4, p < 0.05) (Figure 8A). In the presence of NPY, stimulated cells displayed a reduction of FcγRIIA expression levels, indicating that NPY may inhibit phagocytosis by decreasing the level of receptors on the plasma membrane (mean_LPS+NPY _= 97.72 ± 6.30%; mean_IL-1β+NPY _= 93.81 ± 6.18%; n = 4, p < 0.05).

In addition, using immunocytochemistry, we observed that both LPS and IL-β increased FcγRIIA expression levels in microglial cells. Moreover, NPY treatment significantly reduced FcγRIIA expression on either LPS- or IL-β-stimulated cells, to levels similar to untreated cells (Figure 8B).

## Discussion

Microglial cells play a pivotal role in immunosurveillance in the CNS, acting as resident macrophages in the brain parenchyma [1,7,26,27]. In recent years, increasing evidence has suggested that NPY is an important regulator of immune system function, with different results obtained concerning the role of this peptide in phagocytosis [10,28-32]. In our study, we used an experimental model of microglial phagocytic activity induced by lipopolysaccharide (LPS) to evaluate the role of NPY on Fc receptor-mediated phagocytosis. Microglial cells are constantly prowling the brain environment and are able to efficiently identify invading pathogens by expressing a vast array of pattern recognition receptors, such as Toll-like receptors (TLRs) [1,2]. Among the different members of the TLR family, TLR4 is the best characterized. TLR4 recognizes LPS, a component of the outer membrane of Gram-negative bacteria [33]. Importantly, LPS-induced phagocytosis in macrophages requires TLR4 signaling since downregulation of TLR4 expression by RNAi significantly compromises the phagocytic activity of these cells [34]. Accordingly, we show that LPS significantly enhances microglial Fc receptor-mediated phagocytosis while NPY, *via *Y_1 _receptor activation, inhibits this effect. Moreover, we observed that LPS-induced phagocytosis of IgG-opsonized beads is a process that occurs with the involvement of IL-1β and downstream activation of the p38 mitogen-activated protein kinase (MAPK) pathway.

In light of our previous results, showing involvement of interleukin-1β (IL-1β) in LPS-stimulated microglial cell activation and motility, we proposed to uncover a role for IL-1β in LPS-stimulated phagocytosis. Accordingly, LPS and ATP co-administration stimulated phagocytosis and this effect was abolished by IL-1ra treatment, suggesting that the effect of LPS is, at least in part, mediated by IL-1β. Interestingly, we would like to emphasize that IL-1β is vital for resolving bacterial infection caused by various pathogens [35,36]. Bacterial infection results in an increased release of IL-1β, which enhances phagocytic cell recruitment to infection sites [36-38]. Moreover, during LPS-induced phagocytosis, the production of the endogenous anti-inflammatory cytokine IL-1ra is increased to balance possible cytotoxic effects of IL-1β on neighboring tissues [39].

*In vitro *studies have described a role for NPY in the modulation of various functions of macrophages, such as adherence, chemotaxis, phagocytosis and superoxide anion production [32,40-43]. Furthermore, we have also identified a role for NPY in inhibition of nitric oxide production and motility/migration by microglial cells [9,16]. In the present study, we show that NPY inhibits IL-1β-induced phagocytosis *via *Y_1 _receptor activation. In agreement with our results, NPY has been shown to decrease phagocytosis in older mice. Interestingly, in these animals the release of IL-1β from peritoneal macrophages was higher than that observed in younger animals, and NPY also inhibited this effect [32]. The role of NPY in regulation of phagocytosis seems to depend also upon the particular pathogen studied and their mechanisms of replication. *In vitro *studies have shown that NPY inhibits engulfment of *Leishmania major *by a monocyte/macrophage murine cell line [43]. Since infection of phagocytes is a crucial step for replication of *Leishmania major*, inhibiting phagocytosis results in a protective action [44]. Moreover, the effect of NPY may vary according to other parameters (e. g. concentration, target cell, stimulus) and depends on interactions between different cell types [30-32,45,46]. Recognition of pathogen-associated molecular patterns triggers TLR signaling through myeloid differentiation primary response gene 88 (MyD88) adapter molecule and activation of mitogen-activated protein kinases (MAPKs) and NF-κB [47]. Furthermore, p38 MAPK signaling has been implicated in phagocytosis carried out by murine macrophages [48] and Drosophila hemocytes [49]. Since cytoskeleton remodeling is vital for cell phagocytosis, p38 could be a putative molecular target to discern which signaling pathways are involved in this process. There are several reports implicating p38 activation in phagocytosis, either as a consequence of phagocytosis or as a necessary step to initiate this process [48-50]. In fact, Blander and colleagues have shown that the use of selective p38 inhibitors impairs the ability of macrophages to phagocyte *E. coli *[51]. Moreover, upon activation, phosphorylated p38 translocates to the nucleus and phosphorylates MAPK-activated protein kinase 2 (MK2) [52]. Cells deficient in MK2 are unable to regulate actin reorganization and, therefore, to form membrane protrusions [53]. MK2 modulation of phagocytosis may occur through the small heat shock protein HSP25/27, since it regulates actin polymerization [54].

Phagocytosis can be triggered by different signals that are recognized by different receptors, triggering different signaling cascades. In our study, we focused on the role of NPY on Fc receptor-mediated phagocytosis, the main phagocytic process occurring in macrophages, a cell type that shares close functional and morphological resemblances to microglia. In fact, NPY reduced expression of Fc receptor on the cell surface of stimulated microglia, thereby restraining the phagocytic capacity of these cells under inflammatory challenge. In this regard, NPY may modulate the phagocytic process in order to maintain microglial activity close to a physiological resting/surveying state, even in the presence of a stimulatory/activating agent.

However, we should emphasize that our findings are limited to the modulation of Fc receptor-mediated phagocytosis. Nevertheless, our data further highlight the putative anti-inflammatory and anti-phagocytic role of NPY, and broaden our knowledge of the therapeutic value of this neuropeptide in the treatment of CNS disorders.

## Conclusions

In an inflammatory context, microglial cells become strongly activated through a process that involves downstream p38 MAPK and HSP27 phosphorylation, and increase their ability to phagocytose. In our study, NPY acting through Y_1 _receptors significantly restrained microglial activation, thereby inhibiting LPS-induced Fc receptor-mediated phagocytosis. Under physiological conditions, resting-like cells show substantially low levels of phosphorylated p38 MAPK and HSP27, thus presenting limited phagocytic ability. The role of NPY in the regulation of microglial cell phagocytosis is promising since this neuropeptide demonstrates a strong inhibitory action on activated microglia-related responses. While scientific research is commonly conducted towards the regulation or improvement of microglial phagocytosis, which appears limited or dysfunctional in chronic neurodegenerative diseases and ageing [55], our findings could potentially lead to the development of new therapeutic targets aiming at the restriction of phagocytosis in pathological conditions occurring in the brain.

## List of abbreviations

ATP: adenosine triphosphate; IL-1β: interleukin-1beta; IL-1ra: interleukin-1 receptor antagonist; LPS: lipopolysaccharide; MAPK: mitogen-activated protein kinase; NPY: neuropeptide Y; SB239063, p38 inhibitor; TLR4: Toll-like receptor 4.

## Competing interests

The authors declare that they have no competing interests.

## Authors' contributions

RF carried the phagocytosis assays, western blotting and immunocytochemistry studies, performed the statistical analysis and wrote the manuscript. TS participated in the phagocytosis assays and western blotting studies. MV participated in the phagocytic cup assays. LC participated in the acquisition of confocal microscopy images. LB participated in the design and coordination of the study and provided financial support. JM and OV conceived the study, participated in its design, provided financial support and coordinated the project. All authors read and approved the manuscript.
